# Effect of food intake on 92 oncological biomarkers by the Proseek Oncology II panel

**DOI:** 10.1186/s13104-019-4237-9

**Published:** 2019-04-02

**Authors:** Magnus Dencker, Ola Björgell, Joanna Hlebowicz

**Affiliations:** 1 Department of Medical Imaging and Physiology, Skåne University Hospital, Lund University, 205 02 Malmö, Sweden; 2 Department of Clinical Sciences, Division of Medicine, Skåne University Hospital, Lund University, Malmö, Sweden

**Keywords:** Proseek Oncology II, Olink

## Abstract

**Objective:**

To evaluates the effect of food intake on 92 oncological biomarkers to evaluate whether the timing of blood sampling could be relevant. Twenty-two healthy subjects were investigated. A total of 92 biomarkers were measured before a standardised meal as well as 30 and 120 min afterwards with the Proseek Multiplex Oncology II kit.

**Results:**

The levels of 6 biomarkers decreased significantly (P < 0.001) 30 min after food intake, and 4 biomarkers remained decreased (P < 0.001) 120 min after food intake. One biomarker was significantly increased (P < 0.001) at both 30 and 120 min after food intake. Some changes were less than 10%. Those biomarkers that showed a difference of more than 10% include: Granzyme H (13%), Methionine aminopeptidase 2 (14%), Secretory carrier-associated membrane protein 3 (39%), FAS-associated death domain protein (41%), and Pancreatic prohormone (79%). This study shows that food intake has a very modest effect on 92 different oncological biomarkers.

*Trial registration* National Library of Medicine trial registration number NCT01027507 (retrospectively registered on December 8, 2009)

## Introduction

Cancer is major cause of death and disability in the world and its prevalence is increasing [1]. The ongoing identification of novel biomarkers is critical to facilitating early detection of oncological disease. The timing of blood sampling, however, could be relevant as food digestion is known to have hemodynamic and metabolic effects [2–7]. In this study, we investigate whether certain biomarkers are affected by food intake. We have previously reported the influence of food intake on biomarkers analysed with the Proseek Multiplex CVD III, Proseek Multiplex CVD II and Proseek Multiplex Neurology I kits, and now advance our efforts with the Proseek Oncology II panel [8–10]. In this study, we assess the effect of food intake on 92 emerging oncological biomarkers. To our knowledge, this is the first study to do this.

## Main text

### Materials and methods

#### Study population

The trial was registered retrospectively at the National Library of Medicine (trial registration number NCT01027507, retrospectively registered on December 8, 2009). The study was approved by the Regional Ethical Review Board in Lund, Sweden. All subjects gave their written informed consent. The study investigated 22 healthy Caucasians (11 male and 11 female aged 25.9 ± 4.2 years). This is an exploratory study, first of its kind, and the data for a power calculation were not available. The data from the present study could be used for power calculations in future studies. None of the subjects took cardiovascular medication, showed symptoms of cardiovascular disease, or had a history of cardiovascular or other chronic disease. The subjects were examined between 7.30 and 11.00 after a minimum 8-hour fast. Their height and weight was measured and body mass index (BMI) calculated. The subjects then consumed a standardised meal consisting of 300 g rice pudding (AXA Goda Gröten Risgrynsgröt; Lantmännen AXA, Järna, Sweden). The total caloric value of the meal was 330 kcal: 10% from protein (9 g), 58% from carbohydrates (48 g), and 32% from fat (12 g).

#### Blood samples

We collected plasma samples in EDTA test tubes before the meal, 30 min after the meal, and 120 min after the meal. We then froze the samples. No subject consumed any beverage during the experiment. We excluded one blood sample from the analysis, taken 30 min after the meal, as it was defective. The 92 biomarkers were then evaluated at the Olink laboratory, blinded to all other data, in Uppsala using the Proximity Extension Assay technique with the Proseek Multiplex Oncology II 96 × 96 reagents kit (Olink Bioscience, Uppsala, Sweden), as described previously [11, 12]. Data are displayed as arbitrary units (AU). Values can be recalculated to actual concentrations using transformation algorithms on the Olink Bioscience website (http://www.olink.com). The conversion is just an estimate, however, and does not provide exact values. The 92 biomarkers analysed included (intra-assay variation and inter-assay variation, as supplied by the manufacturer): Alpha-taxilin (7%,14%), Vascular endothelial growth factor A (8%,13%), Carboxypeptidase E (9%,16%), Kallikrein-13 (7%,16%), Carcinoembryonic antigen-related cell adhesion molecule 1 (5%,13%), Mesothelin (7%,21%), Tumor necrosis factor ligand superfamily member 13 (8%,13%), Pro-epidermal growth factor (9%,11%), Tumor necrosis factor receptor superfamily member 6B (9%,13%), Syndecan-1 (8%,15%), TGF-beta receptor type-2 (8%,15%), Interleukin-6 (8%,12%), CD48 antigen (7%,14%), Secretory carrier-associated membrane protein 3 (8%,15%), T-lymphocyte surface antigen Ly-9 (6%,14%), Interferon gamma receptor 1 (7%,15%), Integrin alpha-V (5%,13%), TNF-related apoptosis-inducing ligand (8%,12%), Kallikrein-11 (8%,14%), Glypican-1 (8%,14%), Tissue factor pathway inhibitor 2 (9%,13%), Kallikrein-8 (7%,14%), Vascular endothelial growth factor receptor 2 (6%,19%), Ly6/PLAUR domain-containing protein 3 (6%,13%), Podocalyxin (5%,16%), Protein S100-A4 (7%,14%), Insulin-like growth factor 1 receptor (8%,15%), Receptor tyrosine-protein kinase erbB-2 (7%,16%), Receptor tyrosine-protein kinase erbB-3 (6%,16%), Stem cell factor (7%,14%), SPARC (5%,11%), Granzyme H (8%,16%), Transforming growth factor alpha (8%,14%), Furin (7%,16%), Protein CYR61 (9%,15%), Kallikrein-14 (9%,15%), FAS-associated death domain protein (7%,13%), Methionine aminopeptidase 2 (7%,12%), Nectin-4 (8%,15%), Tumor necrosis factor ligand superfamily member 6B (9%,13%), Ephrin type-A receptor 2 (8%,15%), Integrin beta-5 (7%,13%), Galectin-1 (5%,15%), Seizure 6-like protein (7%,15%), Transmembrane glycoprotein NMB (5%,16%), Carbonic anhydrase 9 (8%,15%), Melanoma-derived growth regulatory protein (7%,12%), Cathepsin L2 (9%,11%), CD27 antigen (7%,13%), Xaa-Pro aminopeptidase 2 (6%,15%), Receptor tyrosine-protein kinase erbB-4 (6%,15%), Hepatocyte growth factor (8%,15%), Disintegrin and metalloproteinase domain-containing protein 8 (7%,16%), 5′-nucleotidase (7%,23%), Cyclin-dependent kinase inhibitor 1 (7%,12%), Delta-like protein 1 (8%,15%), Midkine (10%,15%), Tyrosine-protein kinase ABL1 (8%,18%), Fibroblast growth factor-binding protein 1 (6%,13%), Toll-like receptor 3 (8%,16%), Tyrosine-protein kinase Lyn (6%,13%), Proto-oncogene tyrosine-protein kinase receptor Ret (8%,17%), Vimentin (9%,15%), Tumor necrosis factor receptor superfamily member 19 (8%,15%), Cornulin (9%,17%), T cell leukemia/lymphoma protein 1A (9%,15%), CD160 antigen (8%,17%), Tumor necrosis factor receptor superfamily member 4 (8%,15%), MHC class I polypeptide-related sequence A and B (6%,17%), WNT1-inducible-signaling pathway protein 1 (8%,15%), VEGF-co regulated chemokine 1 (9%,20%), Pancreatic prohormone (10%,16%), Protein S100-A11 (6%,14%), Amphiregulin (9%,12%), Endothelial cell-specific molecule 1 (10%,15%), C-type lectin domain family 4 member K (8%,14%), ICOS ligand (6%,15%), WAP four-disulfide core domain protein 2 (7%,13%), C-X-C motif chemokine 13, (6%,11%), Mothers against decapentaplegic homolog 5 (7%,13%), A disintegrin and metalloproteinase with thrombospondin motifs 15 (8%,15%), CD70 antigen (7%,16%), R-spondin-3 (10%,13%), Folate receptor gamma (8%,13%), Carcinoembryonic antigen-related cell adhesion molecule 5 (5%,13%), Vascular endothelial growth factor receptor 3 (6%,14%), Mucin-16 (8%,15%), Wnt inhibitory factor 1 (9%,15%), Granzyme B (9%,14%), Fc receptor-like B (8%,14%), Annexin A1 (8%,16%), Folate receptor alpha (8%,15%). One sample for R-spondin-3 and one sample for Fc receptor-like B failed to reach detection levels. In these cases the values were set at the detection level.

#### Statistical analysis

Data are presented as mean ± standard deviation (SD). Statistical analyses were carried out using Statistica 12 (StatSoft Inc, Tulsa, OK, USA). Comparison between fasting values versus 30 and 120 min after food intake for any given biomarker was analysed for significance with the Wilcoxon matched pairs test. Statistical significance was set at P < 0.001 to counteract the problem of multiple comparisons.

### Results and discussion

Six biomarkers showed a significant decrease in levels (P < 0.001) 30 min after food intake, and of those, 4 remained significantly decreased (P < 0.001) 120 min after food intake (Table 1). One biomarker (Pancreatic prohormone) showed a significant increase (P < 0.001) both 30 min and 120 min after food intake. The changes were most often less than 10%. Some biomarkers showed a difference of more than 10%: Granzyme H (13%), Methionine aminopeptidase 2 (14%), Secretory carrier-associated membrane protein 3 (39%), FAS-associated death domain protein (41%), and Pancreatic prohormone (79%). A summary of our results can be found in Table 2.

**Table 1:** Subjects’ anthropometric characteristics. Values are mean ± SD

Variable	
Sex (male/female)	11/11
Body mass (kg)	69 ± 10
Height (cm)	177 ± 8
BMI (kg/m^2^)	21.8 ± 2.2
BSA (m^2^)	1.8 ± 0.2

*BMI* body mass index, *BSA* body surface area

**Table 2:** Summary of findings for 92 biomarkers before, and 30 and 120 min after a standardised meal

Variable	Fasting (*n* = 22)	30 min after food intake (*n* = 21)	120 min after food intake (*n* = 22)
Alpha-taxilin	1.97 ± 0.37	1.84 ± 0.42	1.58 ± 0.47
Vascular endothelial growth factor A	9.72 ± 0.53	9.64 ± 0.56	9.64 ± 0.62
Carboxypeptidase E	2.43 ± 0.47	2.36 ± 0.43*	2.24 ± 0.62
Kallikrein-13	3.27 ± 0.39	3.20 ± 0.48	3.22 ± 0.42
Carcinoembryonic antigen-related cell adhesion molecule 1	6.75 ± 0.15	6.73 ± 0.15	6.73 ± 0.17
Mesothelin	4.61 ± 0.53	4.62 ± 0.54	4.67 ± 0.53
Tumor necrosis factor ligand superfamily member 13	7.19 ± 0.28	7.10 ± 0.28	7.10 ± 0.34
Pro-epidermal growth factor	10.74 ± 0.24	10.13 ± 0.63*	9.73 ± 0.70*
Tumor necrosis factor receptor superfamily member 6B	3.49 ± 0.49	3.51 ± 0.46	3.42 ± 0.72
Syndecan-1	6.24 ± 0.47	6.17 ± 0.50	6.23 ± 0.57
TGF-beta receptor type-2	6.58 ± 0.32	6.47 ± 0.30	6.53 ± 0.36
Interleukin-6	1.03 ± 1.16	1.01 ± 1.23	1.81 ± 1.20
CD48 antigen	6.07 ± 0.27	5.99 ± 0.26	6.03 ± 0.40
Secretory carrier-associated membrane protein 3	1.67 ± 0.57	1.25 ± 0.47	1.02 ± 0.36*
T-lymphocyte surface antigen Ly-9	4.58 ± 0.29	4.52 ± 0.28	4.58 ± 0.42
Interferon gamma receptor 1	3.52 ± 0.32	3.48 ± 0.33	3.50 ± 0.28
Integrin alpha-V	2.28 ± 0.25	2.27 ± 0.26	2.27 ± 0.20
TNF-related apoptosis-inducing ligand	6.83 ± 0.43	6.82 ± 0.40	6.86 ± 0.49
Kallikrein-11	4.95 ± 0.40	4.97 ± 0.38	4.77 ± 0.37
Glypican-1	3.83 ± 0.40	3.79 ± 0.38	3.82 ± 0.33
Tissue factor pathway inhibitor 2	6.36 ± 0.43	6.32 ± 0.40	6.46 ± 0.41
Kallikrein-8	6.27 ± 0.34	6.24 ± 0.34	5.98 ± 0.40
Vascular endothelial growth factor receptor 2	6.38 ± 0.18	6.34 ± 0.18	6.37 ± 0.20
Ly6/PLAUR domain-containing protein 3	3.59 ± 0.28	3.56 ± 0.30	3.57 ± 0.48
Podocalyxin	2.98 ± 0.19	2.99 ± 0.18	3.02 ± 0.18
Protein S100-A4	3.64 ± 0.41	3.63 ± 0.52	3.40 ± 0.49
Insulin-like growth factor 1 receptor	2.92 ± 0.29	2.83 ± 0.32	2.82 ± 0.33
Receptor tyrosine-protein kinase erbB-2	6.31 ± 0.24	6.26 ± 0.26	6.32 ± 0.38
Receptor tyrosine-protein kinase erbB-3	7.19 ± 0.24	7.15 ± 0.21	7.19 ± 0.24
Stem cell factor	8.76 ± 0.32	8.80 ± 0.28	8.79 ± 0.29
SPARC	5.88 ± 0.17	5.85 ± 0.18	5.83 ± 0.16
Granzyme H	2.43 ± 0.56	2.11 ± 0.55*	2.18 ± 0.76
Transforming growth factor alpha	2.78 ± 0.49	2.70 ± 0.58	2.60 ± 0.50
Furin	3.07 ± 0.36	2.96 ± 0.38	3.12 ± 0.41
Protein CYR61	4.27 ± 0.53	4.23 ± 0.51	4.33 ± 0.65
Kallikrein-14	5.46 ± 0.65	5.31 ± 0.67	5.19 ± 0.70
FAS-associated death domain protein	2.06 ± 0.77	1.66 ± 0.73	1.22 ± 0.52*
Methionine aminopeptidase 2	2.85 ± 0.87	2.79 ± 0.83*	2.44 ± 0.61*
Nectin-4	5.52 ± 0.35	5.40 ± 0.32*	5.25 ± 0.49
Tumor necrosis factor ligand superfamily member 6	8.72 ± 0.36	8.67 ± 0.37	8.74 ± 0.44
Ephrin type-A receptor 2	1.11 ± 0.30	1.08 ± 0.32	1.06 ± 0.31
Integrin beta-5	6.33 ± 0.25	6.27 ± 0.26	6.28 ± 0.28
Galectin-1	4.76 ± 0.21	4.76 ± 0.21	4.68 ± 0.31
Seizure 6-like protein	4.46 ± 0.24	4.46 ± 0.28	4.45 ± 0.29
Transmembrane glycoprotein NMB	5.38 ± 0.16	5.36 ± 0.19	5.37 ± 0.18
Carbonic anhydrase 9	1.66 ± 0.56	1.77 ± 0.55	1.69 ± 0.75
Melanoma-derived growth regulatory protein	9.32 ± 0.26	9.31 ± 0.26	9.26 ± 0.28
Cathepsin L2	2.80 ± 0.37	2.59 ± 0.30*	2.59 ± 0.44
CD27 antigen	7.52 ± 0.34	7.46 ± 0.34	7.50 ± 0.49
Xaa-Pro aminopeptidase 2	5.96 ± 0.47	5.90 ± 0.47	5.98 ± 0.53
Receptor tyrosine-protein kinase erbB-4	4.37 ± 0.23	4.31 ± 0.25	4.36 ± 0.34
Hepatocyte growth factor	6.15 ± 0.54	6.06 ± 0.50	6.05 ± 0.52
Disintegrin and metalloproteinase domain-containing protein 8	4.10 ± 0.33	4.00 ± 0.29	3.96 ± 0.39
5′-Nucleotidase	9.05 ± 0.31	8.95 ± 0.28	9.00 ± 0.34
Cyclin-dependent kinase inhibitor 1	0.52 ± 0.49	0.42 ± 0.48	0.21 ± 0.56
Delta-like protein 1	8.60 ± 0.31	8.57 ± 0.27	8.59 ± 0.34
Midkine	5.11 ± 0.48	4.82 ± 0.63	4.80 ± 0.72
Tyrosine-protein kinase ABL1	1.56 ± 0.68	1.31 ± 0.69	1.13 ± 0.69
Fibroblast growth factor-binding protein 1	6.21 ± 0.26	6.19 ± 0.34	6.10 ± 0.37
Toll-like receptor 3	4.68 ± 0.57	4.64 ± 0.62	4.68 ± 0.68
Tyrosine-protein kinase Lyn	− 0.06 ± 0.19	− 0.15 ± 0.21	− 0.19 ± 0.25
Proto-oncogene tyrosine-protein kinase receptor Ret	4.30 ± 0.47	4.24 ± 0.45	4.29 ± 0.43
Vimentin	3.68 ± 0.37	3.49 ± 0.47	3.32 ± 0.49
Tumor necrosis factor receptor superfamily member 19	3.95 ± 0.41	3.90 ± 0.36	3.81 ± 0.54
Cornulin	5.44 ± 1.16	5.46 ± 1.18	5.51 ± 0.99
T-cell leukemia/lymphoma protein 1A	3.89 ± 0.82	3.78 ± 0.82	3.65 ± 0.90
CD160 antigen	4.61 ± 0.35	4.55 ± 0.28	4.51 ± 0.53
Tumor necrosis factor receptor superfamily member 4	2.57 ± 0.40	2.51 ± 0.36	2.51 ± 0.45
MHC class I polypeptide-related sequence A and B	2.60 ± 1.78	2.49 ± 1.80	2.56 ± 1.85
WNT1-inducible-signaling pathway protein 1	4.58 ± 0.60	4.48 ± 0.64	4.51 ± 0.59
VEGF-co regulated chemokine 1	1.69 ± 0.46	1.69 ± 0.51	1.70 ± 0.61
Pancreatic prohormone	2.39 ± 0.79	4.29 ± 0.91*	3.71 ± 1.22*
Protein S100-A11	2.73 ± 0.36	2.62 ± 0.36	2.56 ± 0.46
Amphiregulin	1.28 ± 0.46	1.26 ± 0.37	1.21 ± 0.41
Endothelial cell-specific molecule 1	7.99 ± 0.48	8.02 ± 0.47	8.09 ± 0.52
C-type lectin domain family 4 member K	2.29 ± 0.34	2.22 ± 0.30	2.24 ± 0.45
ICOS ligand	6.13 ± 0.38	6.00 ± 0.46	6.11 ± 0.39
WAP four-disulfide core domain protein 2	6.38 ± 0.32	6.36 ± 0.30	6.32 ± 0.40
C-X-C motif chemokine 13	8.26 ± 0.57	8.20 ± 0.51	8.32 ± 0.60
Mothers against decapentaplegic homolog 5	1.76 ± 0.46	1.80 ± 0.55	1.78 ± 0.56
A disintegrin and metalloproteinase with thrombospondin motifs 15	0.35 ± 0.43	0.45 ± 0.42	0.35 ± 0.52
CD70 antigen	2.84 ± 0.35	2.87 ± 0.38	2.90 ± 0.55
R-spondin-3	− 0.20 ± 0.36	− 0.28 ± 0.36	− 0.07 ± 0.53
Folate receptor gamma	6.51 ± 1.69	6.48 ± 1.75	6.44 ± 1.75
Carcinoembryonic antigen-related cell adhesion molecule 5	1.45 ± 0.62	1.38 ± 0.70	1.48 ± 0.64
Vascular endothelial growth factor receptor 3	5.56 ± 0.25	5.54 ± 0.25	5.51 ± 0.31
Mucin-16	3.07 ± 0.85	3.12 ± 0.78	3.14 ± 0.92
Wnt inhibitory factor 1	5.23 ± 0.47	5.12 ± 0.42	5.16 ± 0.50
Granzyme B	1.91 ± 0.81	1.75 ± 0.86	1.85 ± 0.90
Fc receptor-like B	− 0.39 ± 0.55	− 0.33 ± 0.55	− 0.38 ± 0.57
Annexin A1	1.93 ± 0.43	1.82 ± 0.47	1.69 ± 0.50
Folate receptor alpha	5.83 ± 0.29	5.79 ± 0.28	5.81 ± 0.43

All values are in arbitrary units (Mean ± SD)

* Indicates significant difference (P < 0.001), compared to fasting values

To our knowledge, no study has previously evaluated the effect of food intake on plasma proteins measured by the Proseek Multiplex Oncology II kit. Our results show that of the 92 biomarkers investigated, only 9 were affected by food intake, and the changes were modest: less than 10%. Standardising food intake, therefore, is generally not required when using this kit. There are several exceptions. The greatest changes in observed levels were for Granzyme H (13%), Methionine aminopeptidase 2 (14%), Secretory carrier-associated membrane protein 3 (39%), FAS-associated death domain protein (41%), and Pancreatic prohormone (79%). Our results point to the need to standardise food intake when evaluating these biomarkers.

In our study, we were able to simultaneously measure 92 plasma proteins using the Proseek Multiplex Oncology II kit. The biomarkers were selected because of their oncological relevance. Twenty-one of these biomarkers are defined as cell proliferation, 11 tumor-specific, 11 general oncology, 10 angiogenesis specific, 9 immune response, 8 cell adhesion, 7 exploratory, 6 apoptosis, 5 invasion and metastasis and 4 cell differentiation. This new technology has been used in several studies [13–21], and our investigation adds methodological data to this developing field.

We observed most of the changes 30 min after food intake. A total of 7 biomarkers decreased, and one increased, 30 min after the meal. This could be due to the changes in hemodynamics. We have previously reported a 20% increase in stroke volume and a 28% increase in cardiac output 30 min after food intake in this cohort [4]. There was one obvious exception. Pancreatic prohormone, also known as Pancreatic polypeptide, is secreted by the pancreatic islets of Langerhans and is known to be released by food intake, particularly fat-rich food [22]. Moreover, Pancreatic prohormone has been known to decrease both appetite and food intake [22]. Our finding is not novel concerning postprandial changes of this biomarker. We are not aware of any study that has investigated postprandial levels of Methionine aminopeptidase 2, Secretory carrier associated membrane protein 3, or FAS-associated death domain protein. Granzyme H is part of a group of proteases that are found mainly in cytotoxic immune cells, and has been investigated in a variety of diseases including infectious diseases, multiple sclerosis, large granular lymphocyte leukemia, lymphoma, and breast cancer [23]. Methionine aminopeptidase 2 has been suggested to have an important role in angiogenesis, which is pivotal for the progression of solid tumours [24]. Secretory carrier associated membrane protein 3 functions as a carrier to the cell surface and is also involved in other intracellular protein trafficking, and has to our knowledge only been studied on a cellular or tissue level and our findings may not be relevant [25, 26]. FAS-associated death domain protein has a known role in apoptosis, but has also been implicated in cell proliferation, cell cycle regulation, and cell development. It has also been investigated in squamous cell carcinoma of the head and neck and in non-small cell lung cancer [27, 28].

To our knowledge, only one study has previously assessed the impact of food intake on multiple biomarkers. Jahn et al. evaluated Kallikrein-11, Xaa-Pro aminopeptidase 2 (mAmP), Hepatocyte growth factor, Endothelial cell specific molecule 1, and mucin 16 (Cancer antigen-125) [29]. Their results, for the most part, coincided with ours: food intake showed no effect on biomarker levels. Kallikrein-11, however, was the exception. Jahn et al. found a decrease in Kallikrein-11 levels after food intake, which we did not observe. The variance could be explained by a difference in observation time and/or meal composition.

## Limitations

The limitations of the study include the study population, which consisted of young, healthy Caucasian subjects. Additional studies are recommended in older healthy subjects from different ethnic groups and in patients with disease to determine whether these findings are reproducible. Moreover, the potential interaction effect of different medications should also be considered. Further research is also recommended to evaluate the effect of different diets such as high or low fat [30].

## Abbreviations

BMI: body mass index
AU: arbitrary units
SD: standard deviation
BSA: body surface area
